# Why is There Low Morbidity and Mortality of COVID-19 in Africa?

**DOI:** 10.4269/ajtmh.20-0474

**Published:** 2020-06-01

**Authors:** M. Kariuki Njenga, Jeanette Dawa, Mark Nanyingi, John Gachohi, Isaac Ngere, Michael Letko, C. F. Otieno, Bronwyn M. Gunn, Eric Osoro

**Affiliations:** 1 Washington State University Global Health Program - Kenya, Nairobi, Kenya;; 2 College of Health Sciences, University of Nairobi, Nairobi, Kenya;; 3 Institute of Global Health and Infection, University of Liverpool, Liverpool, United Kingdom;; 4 School of Public Health, Jomo Kenyatta University of Agriculture and Technology, Nairobi, Kenya;; 5 Paul G. Allen School of Global Animal Health, Washington State University, Pullma, Washington

## Abstract

Three months since the detection of the first COVID-19 case in Africa, almost all countries of the continent continued to report lower morbidity and mortality than the global trend, including Europe and North America. We reviewed the merits of various hypotheses advanced to explain this phenomenon, including low seeding rate, effective mitigation measures, population that is more youthful, favorable weather, and possible prior exposure to a cross-reactive virus. Having a youthful population and favorable weather appears compelling, particularly their combined effect; however, progression of the pandemic in the region and globally may dispel these in the coming months.

## INTRODUCTION

**Figure 1.**
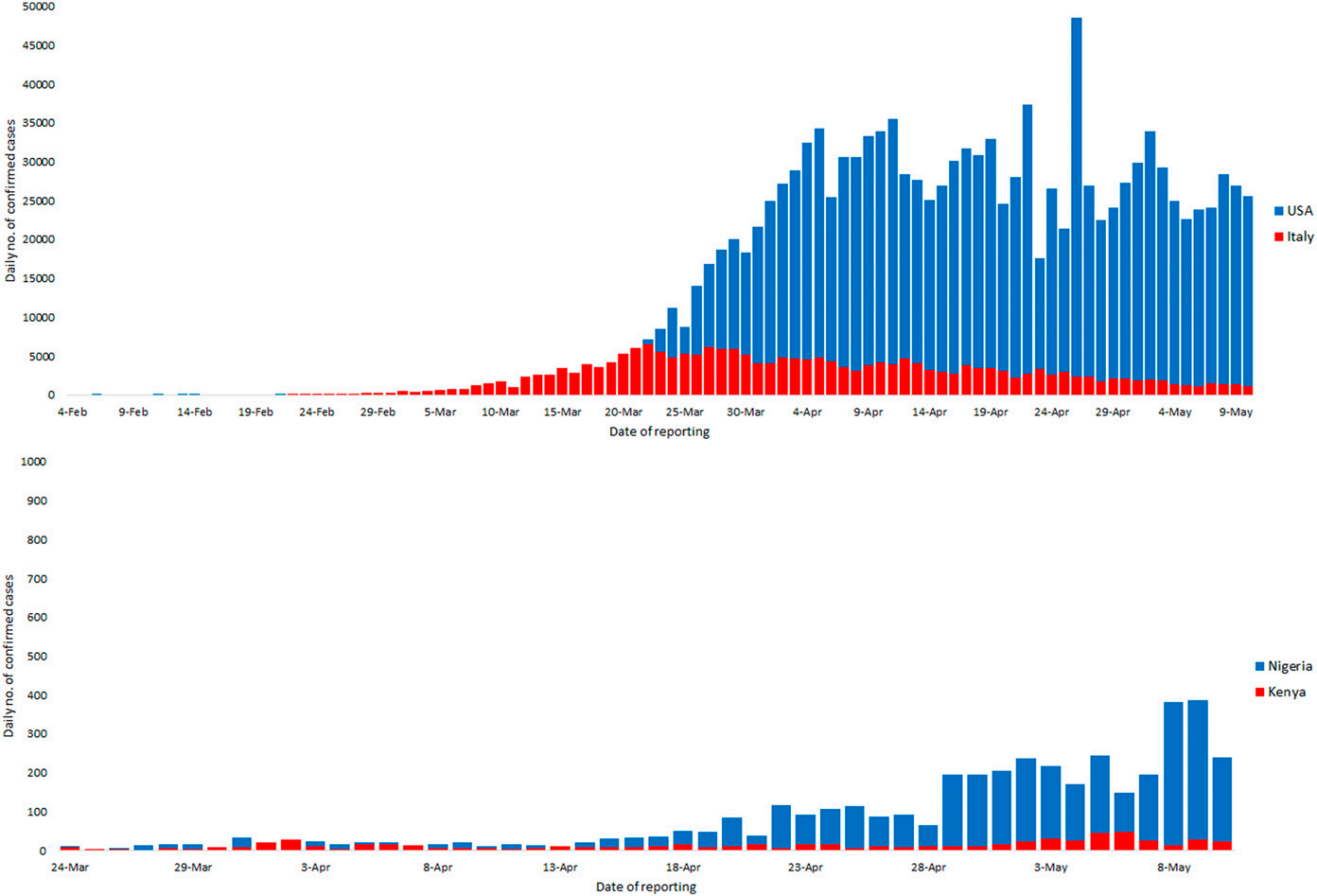
COVID-19 epi curves for the United States and Italy (top) and Nigeria and Kenya (bottom). The *x* axis starts from 2 weeks after the first reported case in the United States (top) and Nigeria (bottom). The different *y* axis scales were used to allow visibility of the low number of cases in Nigeria and Kenya when compared with the United States and Italy. Data used to develop these curves were obtained from publicly available repositories and national health ministries as described in the Data Sources section.

**Figure 2.**
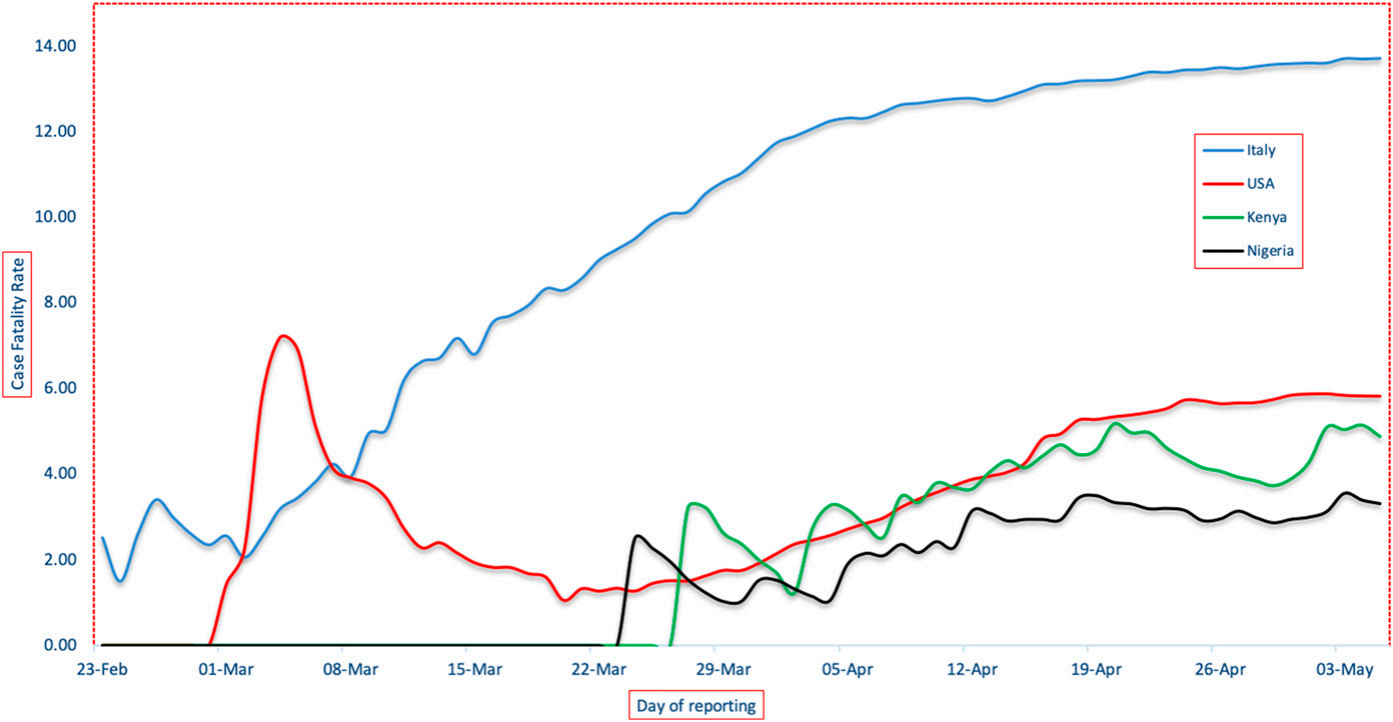
COVID-19 case fatality rate (CFR) for the United States, Italy, Nigeria, and Kenya. Data used to calculate the CFR were downloaded from publicly available repositories and national health ministries as described in the Data Sources section. The limitations to the CFR provided here include the fact that the number of cases (denominator) from each country is dependent on the strength of each country’s surveillance system and may underestimate the actual number of cases because of limitations in testing or those that do not seek medical care due to asymptomatic or mild infections.

COVID-19 is caused by SARS-CoV-2, which was first detected in December 2019 in Hubei Province, China, and declared a public health emergency of international concern on January 30, 2020, and a global pandemic on March 11, 2020, by the WHO.^1^ Unlike recent pandemics, COVID-19 has caused extremely high morbidity (∼5.27 million cases) and significant fatalities (case fatality rate [CFR] ∼6.5%) worldwide, with unprecedented disruption of people’s lifestyles, and unfathomed devastation of global economies. Of the 5.27 million cases reported in more than 200 countries worldwide by May 24, 2020, the Americas accounted for ∼2.42 million with 5.9% fatalities, Europe ∼ 1.81 million with 9.3% fatalities, Asia ∼ 927,000 with 2.9% fatalities, and Africa ∼ 108,000 with 3.0% fatalities, and Oceania ∼ 8,600 cases with 1.5% fatalities. The first COVID-19 case in Africa was reported in Egypt on February 14, and 3 months later, the epidemic curve in the continent remained flatter than that in continental Americas, Europe, and Asia (Figures 1 and 2), and with a lower CFR than the Americas and Europe but comparable to Asia. By May 24, 2020, Nigeria (population ∼ 200 million) had reported 7,526 cases and 221 fatalities (2.9%), whereas Kenya (population ∼ 47 million) had reported 1,192 total cases and 50 fatalities (4.2%).^2^ On the other hand, the United States (population ∼ 328 million) on its fourth month of the pandemic had reported 1,622,670 cases and 97,087 fatalities (6.0%), whereas Italy (population ∼ 60 million) had reported 229,327 cases and 32,735 fatalities (14.1%) (Figures 1 and 2). The higher CFR in Italy may be due to relatively high population density (206 persons/km^2^) of an aging population (median age ∼ 45 years), when compared with either Nigeria with a comparable population density (212 persons/km^2^) but younger population (median age ∼ 18 years), or the United States with comparable population age (median age ∼ 38 years) but lower density (36 persons/km^2^).^3^

We argue that the low number of cases in Africa may not be an artifact of poor surveillance and low testing because an escalating number of COVID-19 cases would be easily detected through reports of pneumonia clusters at local hospitals, which has not been observed. Whereas it is likely that COVID-19 surveillance and testing are weaker in Africa because of limited resources, the high transmissibility of this virus demonstrated in Asia, Europe, and North America (basic reproductive number, *R*_*o*_, of 2–3^4,5^) suggests that local transmission in densely populated cities of Africa such as Lagos or Nairobi would result in clusters of pneumonia at local hospitals. A contrary opinion that SARS-CoV-2 transmission in the continent is comparable to that elsewhere but progression to severe disease is significantly lower remains legitimate. Many public health experts are surprised that the CFR in the region has not soared, given the high burden of HIV/AIDS, tuberculosis, malaria and other infections, and other underlying conditions such as malnutrition and high population density in the urban informal settlements with poor hygiene and sanitation (Figure 2). However, it is worth remembering that the underlying comorbidities associated with severe COVID-19 disease are noncommunicable such as diabetes and asthma, which are less prevalent in Africa.^6–8^ The As of May 24, 2020, the CFR in Africa has remained below the global average and that of Europe and the United States, despite significantly weaker health systems. Here, we reviewed the merits of various hypotheses advanced to explain the relatively low morbidity and mortality of SARS-CoV-2 in Africa.

## HYPOTHESES

The hypotheses we reviewed included low seeding rate, effective mitigation measures, population that is more youthful, favorable weather, and possible preexisting immunity due to prior exposure to other coronaviruses. Other hypotheses advanced to try to explain the phenomenon have fallen apart as the pandemic has progressed. Some experts argue that there were low numbers of SARS-CoV-2 introduced (seeding) into Africa, primarily because of the low volume of air travel to the region.^9^ Many African countries also implemented partial or complete travel restrictions, which further reduced the rate of introduction of imported cases, thus making it easier to identify and isolate initial cases and their contacts, and to limit pockets of transmission. Facing unprecedented collapse in coordinated multilateral response, African countries have demonstrated impressive innovation and resolve in implementing mitigation measures to reduce SARS-CoV-2 spread, largely anchored by the WHO-supported Integrated Disease Surveillance and Response (IDSR) structures and lessons learned from the recurrent Ebola epidemics in the continent.^10^ Nonetheless, we view the two hypotheses as less compelling to explain this initial COVID-19 pandemic trajectory in Africa. As observed, the high transmissibility of SARS-CoV-2 in susceptible populations makes it likely that few introductions would be adequate to trigger a full-blown local epidemic.^4,5^ Importantly, most of the COVID-19 mitigation measures applied in Africa have been less stringent when than those applied in Europe and the United States, in large part because social distancing and lockdowns were not feasible in many parts of Africa because of poverty and overreliance on the informal economic sector for the livelihood most of the people in the continent.^11^

Africa’s more youthful population, with a median age of < 20 years when compared with Europe and the United States (median age > 38 years), may have contributed to the low numbers of severe COVID-19 cases and deaths.^3,12^ This is a plausible argument, even though its contribution may be less because of other pervasive underlying factors such as malnutrition, and risky livelihood and cultural factors brought about by the characteristics of the informal economic sectors they work in, as well as overcrowding within urban settlements. A recent study assessing the impact of population age on COVID-19 fatalities found a standardized mortality ratio, which use age-specific CFRs, that was 4-fold less in Africa when compared to Europe and North America and > 2-fold less when compared to Asia and South America.^13^ Because most young persons infected with SARS-CoV-2 are asymptomatic or have mild symptoms that can be missed by targeted surveillance and testing, the contribution of this factor may be better assessed by conducting well-designed prevalence studies to determine the extent of SARS-CoV-2 infections in various settings (urban, peri-urban, and rural) within the continent.

**Figure 3.**
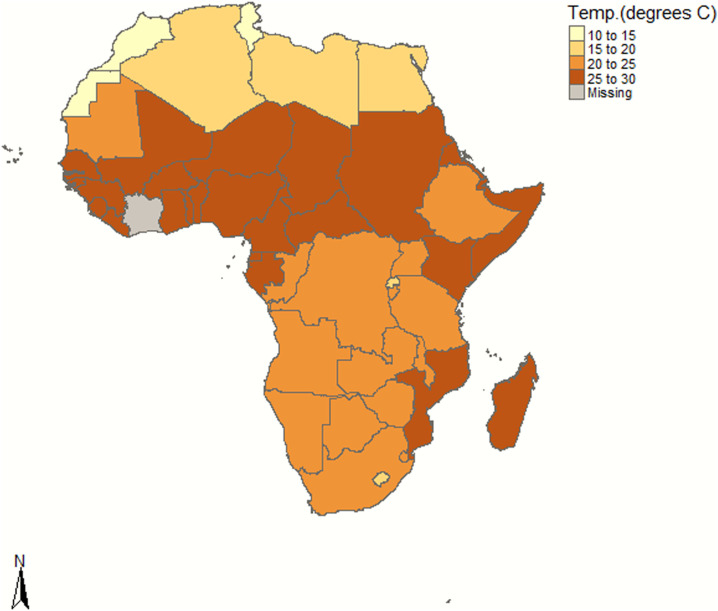
Average daily temperature between December and April over 10 years (2004–2013) in African countries. Data from Côte d’Ivoire were not available.

Africa experiences warmer and drier weather in the December to April season, with average day temperatures > 20°C in the entire sub-Saharan Africa region and more than 30 of the 46 countries of that region averaging > 25°C as shown in Figure 3. Only countries in the northern region including Tunisia, Algeria, Egypt, Libya, and Morocco have day temperatures < 20°C (Figure 3). The sub-Saharan African region has little day temperature variation over the 5 months period and throughout the calendar year, decreasing between May and August but still staying > 20°C in most countries.^11,14^ It is plausible that this warmer weather is decreasing the transmissibility of SARS-CoV-2 in Africa, as has been demonstrated with other respiratory viruses such as influenza.^15^ In the cases of influenza viruses, the high disease burden in winter and colder seasons globally is associated with the increased viability of the virus in cold dry conditions with low levels of sunlight and the tendency of people to spend more time indoors, enhancing spread.^10,11^ Even in equatorial African countries that do not have prolonged seasons with extreme temperatures, influenza cases increase during the cool dry months.^15–17^ It is important to note that other aerosol-transmitted viruses that emerged in the recent past, including SARS-CoV-1 in 2002, highly pathogenic H5N1 influenza virus in 2005, and Middle East respiratory syndrome coronavirus(MERS-CoV) in 2012, caused few cases in Africa.^18–20^ In fact, our ongoing studies on the MERS-CoV, a virus that has continued to cause smaller human outbreaks in more than 27 countries globally, have detected widespread virus transmission in African dromedary camels, the natural reservoir of the virus, but few acute human cases in Kenya and the region (I. Ngere and M. K. Njenga, unpublished data).^21^ A possible exception to this was the 2009 pandemic H1N1 influenza virus, which spread rapidly globally including Africa to establish chronic infections and become part of seasonal influenza viruses.^22^ A recent regression analysis study comparing effect of temperature on the number of COVID-19 cases across the 204 affected countries, exclusive of other possible factors, found countries with higher temperatures reporting lower infections, while those with lower temperatures serving as pandemic hotspots.^23^ Other studies on the effect of weather on SARS-CoV-2 transmission are conflicting. Whereas a field study in Spain found no evidence of reduction in cases during different daily temperature fluctuations, laboratory studies showed low stability of the virus in the environment with titers declining rapidly at temperatures of 23–25°C.^24–27^

Progression of the pandemic in India, Australia, and New Zealand supports the argument that warmer weather plays a positive role in reducing SARS-CoV-2 transmission and disease severity. India, the world’s second most populous country (population ∼ 1.38 billion, density 464/km^2^) with average daytime temperature > 24°C between December and April, is inhabited by a slightly younger population (median age ∼ 29 years). The country reported its first case on January 30, 2020 and by May 22, 2020; it had reported 119,574 cases and 3,600 fatalities (3.0%). Australia (population ∼ 25 million, density 9/km^2^) and New Zealand (population ∼ 5 million, density 18/km^2^), both inhabited by older populations (median age ∼ 38 years), had reported 7,095 cases with 101 fatalities (1.4%) and 1,504 cases with 21 fatalities (1.4%), respectively. By contrast, Brazil (population 209 mill, density 25/km^2^) has warm December to April season, but it had high transmission rate and fatalities, reporting 312,074 COVID-19 cases and 20,112 (6.4%) fatalities by May 22, 2020, the highest in South America perhaps because it has struggled to implement standard mitigation measures to reduce transmission.

The last hypothesis is that a population across Africa has some level of SARS-CoV-2 immunity because of prior exposure to other coronaviruses. As with SARS-COV-2, a spillover of zoonotic coronaviruses into the human population has been recorded several times before, and mounting evidence suggests that other strains closely related to human coronaviruses are circulating within bat populations in Africa and elsewhere.^28–32^ Although a novel outbreak of coronavirus has not been reported in the region, the continuous contact between bats, livestock, and humans in rural Africa may have resulted in exposure to these emergent coronaviruses and development of humoral cross-reactivity.^21^ Antibodies that target conserved epitopes across virus families have been identified in humans, as shown for filoviruses where identification of antibodies that cross-neutralize multiple *Ebolaviruses* resulted in the development of promising pan-*Ebolavirus* therapeutic antibodies.^33–35^ The coronavirus spike protein that mediates cell entry is a target of neutralizing antibodies, and the SARS-COV-2 spike protein demonstrates 85% nucleotide homology to a previously identified bat SARS-like coronavirus and 76% homology to SARS-COV-1.^36–38^ Antibodies mediate antiviral activity through both Fab-mediated neutralization and recruitment of innate immune cells via the antibody Fc domain, and emerging data indicate that antibodies developed against SARS-CoV-1 can cross-neutralize SARS-CoV-2.^39–43^ Such coronavirus cross-reactive antibodies may contribute to a low transmission rate and severe disease associated with SARS-CoV-2 through cross-neutralization and rapid clearance by Fc-mediated innate immune effector functions. In addition, a recent study in the United States detected SARS-CoV-2-reactive CD4+ T cells in up to 60% of SARS-CoV-2 unexposed persons (collected prior to 2019), suggesting pre-existing cross-reactivity with other circulating coronaviruses, which evidently has not be as effective in reducing SARS-CoV-2 transmission given the high transmission in the country.^44^ A comprehensive characterization of humoral and cellular reactivity across coronaviruses in the region may not only provide insight into the COVID-19 trajectory in Africa but also contribute to the ongoing debate on the role and duration of protective immunity against SARS-CoV-2.

Finally, a combination of these factors is likely to contribute even more to the low transmission and reduced disease severity in Africa. In particular, the contrasting trends of the pandemic in countries presented here, and recent studies cited, make the combined effects of warmer weather and youthful population a compelling explanation of the low COVID-19 disease transmission and severity in Africa. The presence of preexisting immunity due to prior exposure to cross-reacting coronaviruses is intriguing but requires further studies. The WHO has warned that Africa could still see increased cases and deaths, as demonstrated in Brazil, in the coming months, a progression that may dispel the hypotheses we deem compelling.

## DATA SOURCES

Data on the current number of cases in each continent were obtained from the Europe CDC (https://www.ecdc.europa.eu/en/geographical-distribution-2019-ncov-cases). Data used to develop the COVID-19 epi curves were accessed from publicly available repositories and national health ministries. The cumulative cases and fatalities for Kenya were extracted from the situation reports (SITREPS) by Emergency Operation Centers under the Ministry of Health (www.health.go.ke), whereas those for Nigeria were extracted from the Nigerian Center for Disease Control website (https://covid19.ncdc.gov.ng). The United States’ daily cases were extracted from the CDC (www.cdc.gov), whereas those for Italy were curated from an interactive web-based dashboard that tracks COVID-19 in real time developed by the John Hopkins University of Medicine.(https://coronavirus.jhu.edu/map.html)^45^ All confirmed cases include presumptive positive cases and probable cases, in accordance with CDC guidelines. The fatality data used to calculate CFRs were downloaded from https://ourworldindata.org/covid-deaths. To confirm reliability of these datasets, we cross-checked with the WHO SITREPS (WHO, 2020) and www.worldometers.info. Temperature data were sourced from publicly available online datasets from Berkeley Earth, covering the months of December to April for 10 years between 2004 and 2013 and averaged by country (https://stat.world/biportal/). A choropleth map was generated in R to show the extracted temperature values for each country covering this period.
